# The Cause and Treatment of Chronic Infections Involving the Teeth—A Discussion of Principles*Read before the Chicago Dental Society, March 15, 1921; reprinted from the *National Dental Journal*, July, 1921.

**Published:** 1921-08

**Authors:** F. B. Moorehead

**Affiliations:** Chicago, Ill.


### THE CAUSE AND TREATMENT OF CHRONIC INFECTIONS INVOLVING THE TEETH—A DISCUSSION OF PRINCIPLES*

BY F. B. MOOREHEAD, D.D.S., CHICAGO, ILL.

Organized dentistry today is a rather formidable affair. It is charged with the responsibility of carrying an enormous overhead, a responsibility that is probably open to criticism when measured in terms of economics. The piling-up process illustrates the relationship between mass and momentum. The general setting of dentistry and of dental practice has become crystallized in point of theory and attitude.

Generally speaking, the function of dentistry is largely terminal. Beginning with a tooth cavity the repair program embraces an extensive remedial detail. The character and extent of repair and reconstruction follows in the wake of tooth destruction by caries and loss of teeth through infection.

Another elaborate program relates to dead pulps of teeth and their sequelae. The management of root-canals is a lively subject.

---

* Read before the Chicago Dental Society, March 15, 1921; reprinted from the *National Dental Journal*, July, 1921.

Circulatory disturbances in the alveolar process and overlying soft tissues, followed by destructive inflammatory processes, call for another comprehensive program of defense. Here, as in the other activities of dentistry, the whole plan of campaign is largely a defensive one. An offensive campaign is another and more vital matter. Dentistry, then, is largely built around two facts: (1) caries of teeth; (2) destructive inflammations of the alveolar process and soft tissues overlying. Organized dental societies, national, state, district and local, the dental journal, the dental college, the manufacturer and purveyor of dental equipment and supplies and the private practitioner, make up the army of defense. Men look at this army and speak confidently of progress. Real progress is not measured by numbers or represented by temporary expedients. The ordinary discussion of dental progress is largely a defensive affair. The premise is dentistry versus medicine. It must be apparent to the thoughtful mind that any attempt at setting up a separate dynasty for dentistry is heresy, sheer iconoclasm. From the most partisan viewpoint, dentistry is a co-ordinate branch of general medicine, and any departure from this principle injures the usefulness of dentistry. This applies both to the recognition and the treatment of disease.

The offensive dental program is limited to occasional and sporadic attempts at original investigation. The general attitude is quite in favor of a defensive program built around the problems of repair and reconstruction by artificial devices, and offers no possible solution of the fundamental problems involved.

The attack is made wholly on the posterior problem. The anterior problem is not seriously considered. The average dental curriculum represents fairly well the general attitude of dentistry. It is divided into two parts, generally called practical and theoretical; we mention the "practical division" first because it is given the major emphasis. It represents the army of defense, and deals wholly with posterior problems. The theoretical divisior is improperly titled. Properly classified, it is the fundamental division, and is made up of the fundamental sciences. This division represents the anterior problem and alone offers any possible progress toward the solution of tooth decay and infection.

At first blush our major argument may sound like a thrust at the body of dentistry. It is not intended as such. Our purpose is to point out the attitude of dentistry and the influence of this attitude on the problem of infection, both local and general. The activity of dentistry is directed at the treatment of the end-results of infection involving the teeth. The anterior problem of dentistry is an intelligent search for the cause of infection, and the establishment of a means of prevention. This would seem to be the whole duty of dentistry. We have been diligent about "mint, anise and cummin," but have sadly neglected the "weightier matters of law." It appears reasonable, therefore, to call attention to the point at which the greater emphasis has been directed, and to suggest a change of emphasis to fundamentals instead of details. It is scarcely necessary, in passing, to observe that the whole matter relates directly to dental education.

Criticism might also be directed at this discussion for the seeming low value placed on so-called mechanical or technical dentistry. This is farthest from our thought. Our thesis is one of principles and not details. As a matter of fact, technical dentistry can not be properly evaluated or applied, except in terms of a biological back ground. This is the difference between an occupation and a scientific profession. Technical dentistry will reach its highest usefulness in the hands of the man who has the scientific conception of its use. From this premise the argument arrives at exactly the same conclusions as the premise chosen. Indeed, the premises are exectly the same in effect. We have predicated dentistry in terms of tradition and practice; not to hold it up for execration, but for the express purpose of discussing dispassionately basic facts.

*The true aim of dentistry is ultimately to eliminate itself.* The vision of the doctor is not of a day when he will have a specific for every ailment and disorder, but rather of the possibility of man exempt from ailments and disorders. The true physician and dentist are not engaged in "doctoring." Their business is to get at the truth and to guide society in the application of truth in the realm of physical well-being. One's professional standing does not depend fundamentally on certain aptitudes, but rather, first, on an attitude of mind, and second, on ability to discharge certain highly specialized functions in society.

Every true profession passes through the experience of determination, whether it shall be simply an occupation or a profession; whether it shall devote itself to the development of certain tricks, devices and superficial efficiencies or that of discovering the universal laws in its domain, and developing harmony between those laws and the human factors. Normally, the movement is from the empirical to the scientific. Any profession or form of functional human service that too long persists in building up the empirical mechanisms will arrive sooner or later in the social discard, and society will find some new way or organization for its function.

In the matter of the organization of certain skills, dentistry has assumed large proportions, but what has it done as a social agency for the study and control of vital processes? The final test to be applied to dentistry as to all similar human activities is service. Is the background of dentistry a workshop or a scientific laboratory?

When the program of patching is converted into a program of prevention the axe will be laid at the root of the tree.

This, briefly stated, represents our first mental reaction to the subject assigned: ''The cause and treatment of chronic infections involving the teeth—a discussion of principles.''

CAUSES

It is freely acknowledged that decay of tooth tissues is the antecedent factor in root-canal infection. It is quite unnecessary in this discussion to review the various theories of dental caries. Whatever the real cause may be, the quality of tooth structure is not fundamental. In the experimental study of scurvy, beri-beri and rickets, interesting circulatory changes in the mouth have been observed. Moore and Jackson report petechial hemorrhages in the pulp among the earliest tissue changes noted in their experimental studies. Actual caries of enamel have been observed by several investigators. Circulatory changes in the gum tissue are another of the early findings. A passive hyperemia followed by infection quickly leads to a suppurative process. This suppurative pericementitis is clinically identical with a similar picture in the human. The teeth become painful, elongated, and are finally lost if the infection is not checked. The interesting observation in these studies is the quick return to normal when the diet is changed to include the so-called vitamines, or fat-soluble and water-soluble substances. The infection disappears and the tissues quickly take on a normal condition without any local treatment.

The chemistry of food substances in relation to the chemistry of metabolism is in all probability fundamental to the problem of infection. It may be highly imaginative to assume that pathogenic microbes will not successfully attack normal tissues. A normal tissue is one adequately supplied with circulating fluids and free from all irritants. We have a feeling that dental caries is a phase of infection susceptibility and that the basic laws governing infection include dental caries. Diet is one of the very serious problems of general medicine and relates as directly to the tissues of the mouth as to other body tissues. Any discussion or appreciation of foods, their use and dangers, presupposes a working knowledge of biochemistry, a subject not widely recognized in the dental curriculum. For that matter, chemistry is generally looked upon as a passing evil—a sort of intellectual travail. Rightly interpreted, chemistry is the most important of the fundamental sciences.

Circulatory changes in the gum tissue and alveolar process are produced by constitutional irritants, excluding, from the discussion, dental technic. These circulatory changes may be relatively classified as transitory and fixed. The transitory form is due to deposits from the saliva and blood, although one may properly question the incidence of so-called ''serumal calculus.''

In the first instance, irritation produced by deposits is mechanical, but may and frequently does prepare the way for incidental local infection. The incidental local infection may, in time, lead to general irritation and harm. The amount of harm is pre-determined by conditions both local and general, and in speaking of local conditions one must bear in mind the responsibility of constitutional influences, the cause being of a general nature, the exhibit local. Ordinarily the tissues correct themselves with the removal of the deposits, although the removal of the deposits has no curative influence on the cause of the deposits.

The second form of circulatory change, largely if not wholly general in character, is a more serious matter. The alveolar process and gum tissue are end-organs. They are not present at birth and usually disappear after the loss of the teeth. Irritants in the circulatory fluids have a serious effect on these end-vessels, setting up a proliferating end-arteritis often leading to obliteration, and even though vessel changes may not be permanent because of collateral circulation, the tissues may be permanently damaged by the proliferation of fixed cells, leading to scar-tissue formation. The alveolar process and gum tissue are necessarily seriously injured, and fall easy prey to infection. Talbot has rightly called this condition gingivitis, although it is more than that in effect. The pericemental membrane is caught between the upper and nether millstones and its destruction determines the future safety and value of the tooth. An appreciation of the seriousness of the circulatory breakdown is the best guide in an attempt at treatment.

Suppuration is not an index of the seriousness or extent of pericemental involvement, nor does it represent a degree of harm constitutionally. A non-suppurative pericementitis may be, and often is, more progressively serious than the suppurative type. Pus formation is incidental, not diagnostic. A general sepsis without pus formation may be fatal; a psoas abscess containing a quart of pus may be relatively harmless. The circulatory changes seen in the soft tissues of the mouth may be more or less localized, or they may be part of a general circulatory change with the heart, liver or kidney as the antecedent factor or factors. If the disturbance is part of a general breakdown, the mouth prognosis is necessarily doubtful. This point is only one of several illustrating the interdependencies of function and repair, and serves to indicate the medical phase of dentistry.

A very interesting observation in these infections is the simultaneous activity of the osteoblasts and osteoclasts in the same field; one cell removing, the other building, tissue. This is sufficient explanation of the incidence of solid teeth involved in an extensive destruction of the alveolar process. A small area of ankylosis will hold a tooth quite solid where the process and membrane have been extensively destroyed. It does not follow, therefore, that because the tooth is solid it may not be hopelessly involved.

*Root-end infection.* Three distinctly chronic conditions follow the introduction of infection into the periapical area: (1) simple granuloma, (2) cyst (infection), (3) liquefaction necrosis. A very large majority of root-end infections probably pass through the root-canal, but not all. The apical area may be permanently injured by the use of arsenic in pulp destruction, or strong chemicals in the treatment of root-canals. Through the use of these irritants the normal circulation is destroyed by the formation of scar tissue. Blood-borne bacteria may localize in such tissue and establish an infection atrium. Any circulatory damage is serious, both in the prevention of infection and insurance of tissue repair. The formation of a cyst at the root-end by the irritation produced by a chronic infection is the least serious of the three forms of root-end involvement.

Second in point of general harm is the simple granuloma. Liquefaction necrosis is the more harmful of the three because of the absence of a limiting membrane and exposure of blood and lymph vessels. Discussing in retrospect for a moment, the question of cause may be epitomized in two groups: (1) root-end infections, (2) circulatory changes in the alveolar process and overlying tissues. The cause in the first group is largely infection through the root-canal following tooth decay. Blood and lymph-borne infections implanted in the apical area when the resistance has been lowered, or largely lost through scar-tissue formation, are brought about by strong chemicals used in pulp destruction or in the treatment of infected root-canals.

The cause in the second group involves a more complicated mechanism. An interlocking local and general circulatory embarrassment is discovered. In one, the cause is both local and general, in the other it is entirely general.

In the first of these, deposits of tartar produce a localized circulatory change. Infection is incidental and clears up when the irritant has been removed. In the second, the circulatory disturbance is fundamental and more or less permanent, depending largely on the condition of the cardiovascular mechanism. Given the two causes mentioned, the condition of tissue injury and loss is simple and to be expected.

The result of injury and loss is quite complex, and the end-result far-reaching and serious.

### TREATMENT

The early, intelligent, successful filling of a tooth cavity is fundamentally economic, and while it is prevention of a secondary type, the results are most wholesome and beneficial. If all tooth cavities could be successfully treated by fillings, the human race would be protected from one of the great sources of harm, and dentistry would be reduced to an irreducible minimum, recognizing tooth decay as a fact. *If the dentist discovers the cause and prevention of caries, he will have served the highest and holiest purpose of dentistry—self-elimination.*

Pending the day of that notable discovery, the logical point of attack is the early filling of cavities. Since intrinsic tooth infection causes the loss of a greater number of teeth than extrinsic infection, and is largely preventible by cavity treatment, the total of dentistry may be reduced more than half, and the harm from infection even more materially reduced by a relatively simple technical therapy —the filling of tooth cavities.

Antecedent to the treatment of cavities is the more fundamental problem of cavity prevention, and this may be accomplished in part by the early care of the teeth of children and by proper diet. This involves dentistry and pediatrics and suggests the greatest interval in medicine—the interval between the physician and the dentist.

In our discussion of the child, the leak is plainly seen; the dentist knows little about diet, and the pediatrician knows little about teeth, yet separate dental and feeding clinics are established. The well-organized children's clinic engages all medical specialties, including dentistry. This is another way of stating that no dental clinic should be set up independently. Any independent clinic ignores the interdependencies of organs, tissues and functions.

The introduction of the child and the care of his teeth further introduces the social aspect of dentistry.

The selfishness of any profession is measured by the extent of the private practice of that profession, and the altruism is measured by its social relationships and activities.

The failure of dentistry in meeting its obligations to society is largely due to the attempt at maintaining isolated clinics. Group practice is economical from the point of time, material, organization and human benefit. A well-trained dentist should be on the staff of every hospital and dispensary. The dental student can not catch the spirit or purpose of general medicine unless he is associated with the hospital and dispensary. The atmosphere of these institutions fixes in his mind the larger and more important physical problems.

Whatever may be done for the adult, the proper care of the child's mouth is the one certain means of establishing the full value of dental service. The intelligent physical care of the child not only discharges a sacred responsibility, but provides an income in human values far beyond the limits of imagination.

*In the final words of this discussion, we have yet to deal with the tooth involved in chronic infections. Our first conclusion is simple; the infection should be removed. If this can be accomplished without the removal of the tooth, the tooth should be saved; if not, the tooth should be removed.*

If this problem is measured entirely in terms of teeth, few will be removed. If measured, however, in terms of physical well-being and usefulness, more will be removed. The tooth is as intimately related to the blood and lymph supply as any other important structure, and must be managed for the good of the larger interests. The tooth which is of greater service than an artificial substitute should be saved. But the imperative demand of tooth service is safety, comfort and function. In meeting this demand the integrity and judgment of the dentist is challenged.

DISCUSSION

*Frederick B. Noyes:* You have heard a clarion call which summons the dental profession to open its eyes and change its point of view. It is true that the dental profession has been and is concerned chiefly with the posterior problem. This attitude begins in the dental school, where the dental student spends four years focusing his attention upon the making of things and the doing of things, the making of fillings, crowns, bridges, etc., the doing of technical performances, and not upon the primary things or the causes of injury. After four years of that kind of training, it is strange, especially when he discovers an overwhelming demand for the performance of these things in an attack upon the posterior problem, that he finds little time for even a superficial consideration of the primary problem. A very large proportion of the members of the medical profession live their professional lives in going to see people get well if they do not kill them, and collect a fee for it, and the attack on the posterior problem of the dentist is on a similar basis. The average dentist makes fillings and crowns, good or bad, and repairs damages nicely or poorly, but does he treat that patient for caries of the teeth? No. He does what the patient asks him to do. In the case of the medical profession the impulse which commands human nature to call the doctor is the fear of death, and with the dental profession the underlying human factor is the pain and the fear of pain. If the dental profession is to focus its attention upon the anterior problem, it must start from some place to change its point of view, I therefore want to indicate two ways in which this has already been done. Those of you who have studied Dr. Black's work and his teachings may recall the logical sequence of it; he taught that caries was an infection, like measles, chicken pox and whooping cough, and the other infectious diseases of childhood, and that the average human being acquires immunity to caries of the teeth as he requires immunity to measles and whooping cough. Therefore, if the teeth can be saved until he acquires that immunity, he will probably retain his denture throughout life. But Dr. Black did not teach you to fill cavities; he taught you to study caries in the mouths of your individual patients, and the dental profession has failed to grasp and apply his teaching because it has been content to fill cavities for patients and not study the individual for the disease caries.

As the essayist called upon you to institute early treatment, so did Dr. Black call upon you for the early recognition of the beginning of caries, in the beginning of the destruction of tooth tissue, and the treatment of it so as to prevent its recurrence. The meaning of extension for prevention as taught by Dr. Black has been grasped by very few in the profession. There is the place where the dental profession must begin to change its point of view. There is the foundation upon which it should have already begun to build and to concentrate its attention regarding the anterior as distinguished from the posterior problem. The old doctor went far. He recognized that there was still another way of attacking the primary problem, that of the prevention of the beginnings of caries, what he termed prophylaxis; not polishing the tooth surfaces, not tooth manicuring, but the prevention of the systemic conditions related to diet and health which made the individual susceptible to the infection of caries.

Is it not strange that more than ten years ago, Dr. Black, in a large hall in the LaSalle Hotel, presented a paper to this society in which he showed that he could produce deposits of calculus upon his own teeth at will and upon the teeth of others; that it was a matter which was related to diet and systemic conditions, and yet have any of you seen any attention paid to it in the last ten years?

There is still another line referred to in the paper which will lead the profession from its present position into the desired atmosphere, and that is the recognition of the social responsibility which the essayist pleaded for—a recognition of the responsibility of the dental profession for the dental care of the community.

As regards the dental care of employees in great establishments, attempts have been made but they have been greatly handicapped, often destroyed in their efficiency, because of conditions which could not be overcome. It is one of the phases of responsibility of the profession which must be worked out. The second is the responsibility of the dental profession for the dental care of the individuals in state institutions. In fact, not until the whole profession through its organization works out a plan and devotes time and energy from private interests will the dental care of the penitentiary, of the reform schools, of the insane asylums, and of all the other state institutions be approximately what it should be. And last, and most important of all, as a final plea of the essayist, is the care of the children in our public schools, the organization of mouth hygiene instruction, etc. We must recognize that the school, under the present civilized community, has taken over the function of the family in very great measures, and only through the schools can we reach all the families of the community. Let us all work and therefore hope for the establishment of a dental clinic for children in Chicago, which shall include all the factors that are of interest to the children. Let us be the pioneers to gather around us and around an institution for children all of the men of the medical profession who are interested in the well-being of the child, and let this institution never stop its exertions until Chicago can point not only to a dental infirmary, but a child infirmary which the dental profession has organized.

